# Neighborhood attention transformer multiple instance learning for whole slide image classification

**DOI:** 10.3389/fonc.2024.1389396

**Published:** 2024-08-29

**Authors:** Rukhma Aftab, Qiang Yan, Juanjuan Zhao, Gao Yong, Yue Huajie, Zia Urrehman, Faizi Mohammad Khalid

**Affiliations:** ^1^ College of Computer Science and Technology (College of Data Science), Taiyuan University of Technology, Taiyuan, Shanxi, China; ^2^ School of Software, North University of China, Taiyuan, Shanxi, China; ^3^ Department of Respiratory and Critical Care Medicine, Sinopharm Tongmei General Hospital, Datong, Shanxi, China; ^4^ First Hospital of Shanxi Medical University, Shanxi Medical University, Taiyuan, Shanxi, China

**Keywords:** attention transformer, whole slide images, multiple instance learning, lung cancer, weakly supervised learning

## Abstract

**Introduction:**

Pathologists rely on whole slide images (WSIs) to diagnose cancer by identifying tumor cells and subtypes. Deep learning models, particularly weakly supervised ones, classify WSIs using image tiles but may overlook false positives and negatives due to the heterogeneous nature of tumors. Both cancerous and healthy cells can proliferate in patterns that extend beyond individual tiles, leading to errors at the tile level that result in inaccurate tumor-level classifications.

**Methods:**

To address this limitation, we introduce NATMIL (Neighborhood Attention Transformer Multiple Instance Learning), which utilizes the Neighborhood Attention Transformer to incorporate contextual dependencies among WSI tiles. NATMIL enhances multiple instance learning by integrating a broader tissue context into the model. Our approach enhances the accuracy of tumor classification by considering the broader tissue context, thus reducing errors associated with isolated tile analysis.

**Results:**

We conducted a quantitative analysis to evaluate NATMIL’s performance against other weakly supervised algorithms. When applied to subtyping non-small cell lung cancer (NSCLC) and lymph node (LN) tumors, NATMIL demonstrated superior accuracy. Specifically, NATMIL achieved accuracy values of 89.6% on the Camelyon dataset and 88.1% on the TCGA-LUSC dataset, outperforming existing methods. These results underscore NATMIL’s potential as a robust tool for improving the precision of cancer diagnosis using WSIs.

**Discussion:**

Our findings demonstrate that NATMIL significantly improves tumor classification accuracy by reducing errors associated with isolated tile analysis. The integration of contextual dependencies enhances the precision of cancer diagnosis using WSIs, highlighting NATMILs´ potential as a robust tool in pathology.

## 1 Introduction

The examination of tissue biopsy sections, specifically whole slide images (WSIs), yields a substantial amount of phenotypic data and serves as the fundamental basis for the field of cancer pathology (1). Recently, there has been significant advancement in the field of deep learning (DL) techniques (2). These methods have revolutionized the construction of diagnostic machines that exhibit a high level of accuracy. In fact, their performance in tasks related to cancer classification and diagnosis has been seen to be on par with, or even surpass, that of specialists who have undergone extensive training (3). However, to create effective deep neural network (DNN) models for cancer pathology, it has often been necessary to annotate every WSI on a pixel level using thorough ground-truth descriptions based on expert opinions (4). The utilization of slide-level labels in a weakly supervised scenario for training DNN classification models has exhibited remarkable accuracy in classifying test data. This achievement has significant implications for the implementation of adaptable mathematical systems for decision-making in clinical practice, as evidenced by previous studies (5–7).

In the context of cancer histology, DNN models do not process WSIs as single images at a time like regular images. Instead, WSIs are commonly broken into smaller units known as “tiles” that serve as input elements. Using tile-level DL characteristics, the entire WSI and tumor are classified. The Multiple Instance Learning (MIL) framework is used in most weakly supervised WSI classification algorithms to learn the slide-level label from each WSI as a “bag” of tiles. MIL models are permutation invariant, meaning WSI tiles have no specific ordering, which hinders their deployment and the weakly supervised learning paradigm (8).

The motivation behind this work is to address the limitations of current weakly supervised methods, which often overlook the spatial dependencies among WSI tiles. This oversight can lead to false positives and negatives, particularly given the heterogeneous nature of tumors. To overcome this challenge, we propose a novel and efficient hierarchical transformer model called Neighborhood Attention Transformer Multiple Instance Learning (NATMIL).

The novelty of our approach lies in the Neighborhood Attention mechanism, which localizes the Self-Attention operation to the nearest neighbors of each pixel, without relying on a predetermined window adjacent to the pixel. This updated definition permits all pixels to possess a uniform rate of attention, which would otherwise be diminished for edge pixels in zero-padded options. As the size of the neighborhood increases, neighborhood attention exhibits similarities to self-attention and can be considered equivalent to self-attention when the neighborhood reaches its maximum size. Moreover, the utilization of local attention offers the additional benefit of preserving translational equivariance, which sets it apart from blocked and window self-attention mechanisms.

We have devised a method called the Neighborhood Attention Transformer (NAT) that performs competitively. In conclusion, our most significant contributions are as follows:

- Proposing a simple and adaptable sliding window attention mechanism that preserves translational equivariance, approximates self-attention as its span increases, and localizes each pixel’s attention span to its closest neighborhood. We contrast Neighborhood Attention with window self-attention, convolutions, and self-attention in terms of accuracy.
- Introducing a new hierarchical transformer that leverages Neighborhood Attention (NA)’s efficiency, accuracy, and scalability: the Neighborhood Attention Transformer (NAT). We demonstrate its effectiveness on downstream tasks upon classification.

By addressing the spatial dependencies among WSI tiles and introducing a novel attention mechanism, this work aims to significantly improve the accuracy and reliability of cancer pathology models.

## 2 Related work

In the conventional approach, a WSI is commonly partitioned into non-overlapping tiles of a predetermined size. These tiles are subsequently assigned a weak label, determined based on the diagnosis at the slide level, to be utilized as input for a Deep Neural Network (DNN) (9). The MIL formulation allows for the prediction of a WSI label (cancer yes/no, cancer type) to originate either from the tile predictions (5, 10–12) or from a higher-level bag representation arising from the aggregation of the tile features (8, 13–15). The former method is referred to as instance based. The latter method, which makes use of bag embeddings (8, 14), has been shown to perform better in experiments. Recent bag-embedding-based methods (16) use attention mechanisms, which give each tile a score reflecting its importance in the overall WSI-level representation. Most contemporary bag-embedding-based methods include attention mechanisms (16), which award a score to each tile indicating its relative contribution to the overall representation of the WSI. Attention scores facilitate the automated identification of sub-regions that possess significant diagnostic value and provide information for the label at the WSI level (15, 17, 18).

Different attention-based MIL models investigate WSI tissue structure in various ways. Many of them assume that the tiles are unrelated and randomly distributed, which is why they are permutation invariant. Based on this premise, a recent study (13) suggested an attention-based MIL pooling operator that can be taught to automatically compute the bag embedding as the weighted average of all tile features in the WSI. The adoption and modification of this operator have been extensive, with the inclusion of a clustering layer (15, 19, 20) to enhance the acquisition of semantically rich and distinct class-specific features. Nevertheless, operators that are permutation invariant lack the intrinsic ability to capture the structural dependencies that exist between various tiles in the input. For example, the DSMIL method [DSMIL (21)] employs a non-local operator to calculate an attention score for each tile. This value is determined by comparing the feature representation of the tile with that of a crucial tile. Recently, transformer-based designs have been introduced to examine the correlations among the various tiles of a whole-slide image (WSI). These architectures typically employ a learnable position-dependent signal to effectively integrate the spatial information of the picture (22, 23). To optimize for the classification challenge and generate attention scores while concurrently learning the positional embeddings, TransMIL (24) uses a transformer-like architecture. However, transformer-based methodologies might overlook the fundamental biological processes that regulate the spatial organization of the slide.

The Stand-Alone Self-Attention (SASA) (25) technique is considered one of the initial sliding window self-attention patterns. Its primary objective is to substitute convolutions in current convolutional neural networks (CNNs) (26). Striding the feature map extracts key-value pairs like a convolution with zero padding. While accuracy improved, the implementation had high latency despite lower theoretical cost. Sliding window attention, first used in Longformer (27) for language processing, was later used in Vision Longformer (ViL) (28). Although Longformer and ViL’s implementations differed from SASA, they were unable to grow to larger windows and models due to computational overhead. Liu et al. presented Window and Shifted Window (Swin) Attention (29), non-sliding window-based self-attention mechanisms (30) that split feature maps and apply self-attention to each partition individually. Swin Transformer is a pioneering hierarchical vision transformer. The feature maps are pyramid shaped, reducing spatial dimensionality and boosting depth. Swin’s structure is widely employed in CNNs, making it compatible with other networks for downstream tasks like detection and segmentation. At ImageNet-1K classification, Swin outscored DeiT, which utilizes a convolutional teacher. Swin Transformer is the leading approach for object detection on MS-COCO and semantic segmentation on ADE20K. To address the slowness of SASA, Vaswani et al. (31) introduced HaloNet, which employs a new blocked attention pattern. While this modification does violate translational equivariance, the benefits in terms of both performance and memory are acknowledged. Three phases make up HaloNet’s attention mechanism: blocking, haloing, and attention. Blocking input feature maps into non-overlapping subsets creates queries. Next, “haloed” nearby blocks are extracted as keys and values. Attention is then given to extracted queries and key-value pairs. A novel CNN architecture, ConvNeXt, was proposed by Liu et al. (32), inspired by models like Swin. The aforementioned models do not incorporate attention mechanisms; nevertheless, they demonstrate superior performance compared to Swin in several visual tasks.

Our Neighborhood Attention approach localizes the field of response to a window surrounding each query, eliminating the need for additional strategies like Swin’s cyclic shift. We present Neighborhood Attention Transformer, a hierarchy-based transformer-like model using this attention mechanism, and compare its performance to Swin on image classification, object detection, and semantic segmentation.

## 3 Methodology

The NATMIL approach is founded on the premise that the surrounding neighborhood of a tile contains important information on the level of attention allocated to that specific tile by the model. By establishing a parallel between our framework and the process of analyzing a biopsy slide by a pathologist, one might conceptualize the act of zooming in and out of a particular sub-region as a means to comprehensively explore its broader surroundings, so enhancing our understanding of the adjacent micro-environment and tissue.

**Figure 1:**
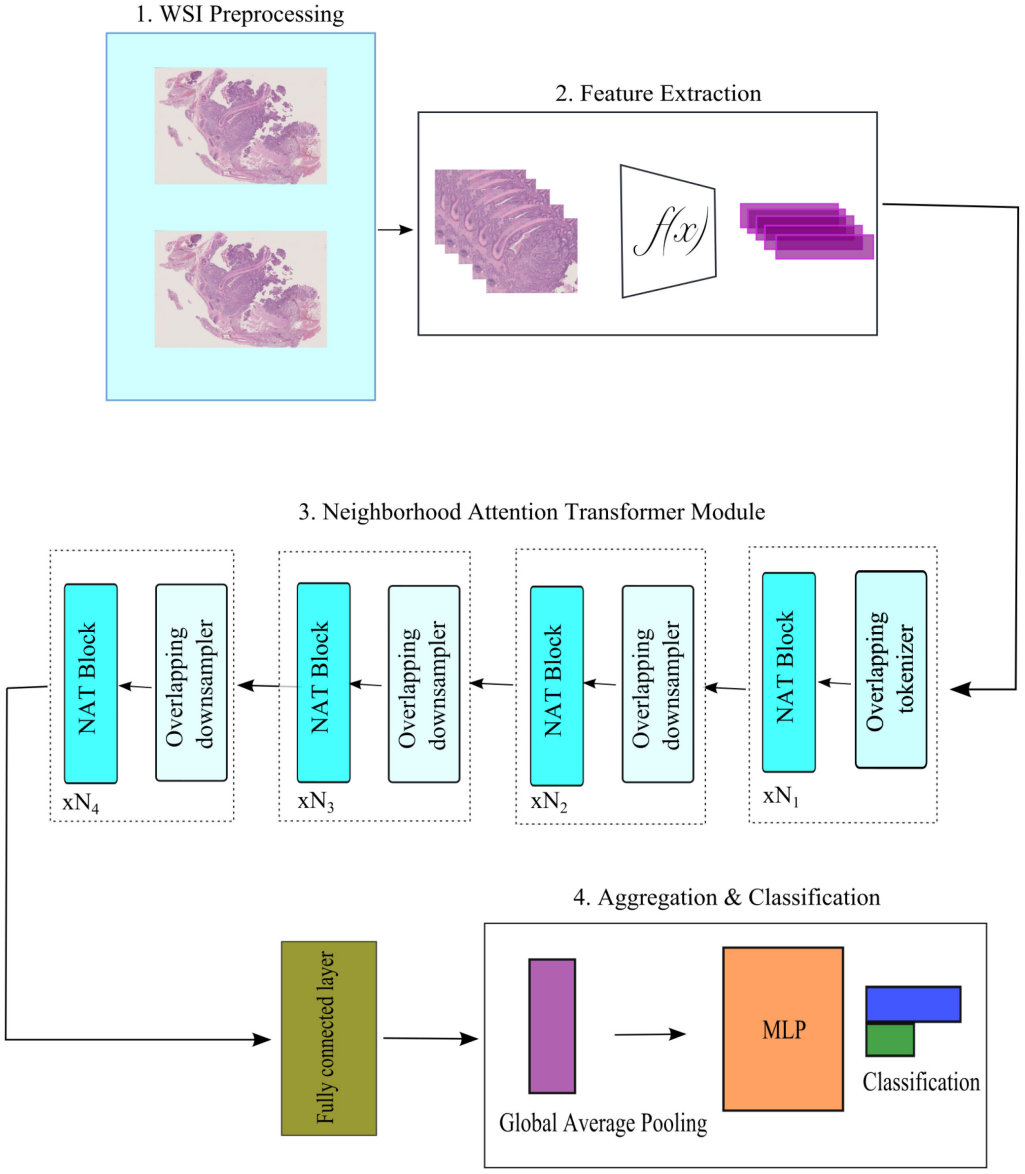
An overview of NATMIL model architecture. At first, preprocessing WSIs separates tissue from background. After splitting the WSIs into 256 × 256 tiles, a pre-trained feature extractor generates 1,024 feature representations for each tile. Tile feature representations function as input for our Neighborhood Attention Transformer module. This module analyzes each patch and its neighbors, creating neighborhood descriptors and calculating attention coefficients. The output layer combines tile-level attention scores from the previous layer to get a slide categorization score.

In NATMIL, the attention score of each tile is recalibrated by combining the attention scores of its surrounding tiles. **Figure 1** provides an overview of the model. It may be broken down into four parts:

1. Each WSI undergoes a preprocessing step in which the tissue area is automatically segmented and divided into several smaller patches.

2. The patch and feature extraction module is composed of a series of convolutional, max pooling, and linear layers. Its purpose is to convert the initial tile input into low-dimensional feature representations. Let $\;H=\left\{h_{1},h_{2},\cdots ,h_{i},\cdots ,h_{N}\right\}$ , where each $h_{i}\in R^{n\times d}$ . Here, d represents the embedding dimensions of a tile, *n* represents the number of tiles inside a WSI, and *N* represents the total number of WSIs.

3. An attention vector of dimension *N* × 1 is produced by a Neighborhood Attention mechanism with a contrastive learning block that incorporates the localizing self-attention to the nearest neighboring pixels.

4. A feature aggregator and classification layer that combines the slide-level prediction and tile-level attention scores produced by the one prior to it.

### 3.1 Feature extractor

**Figure 2:**
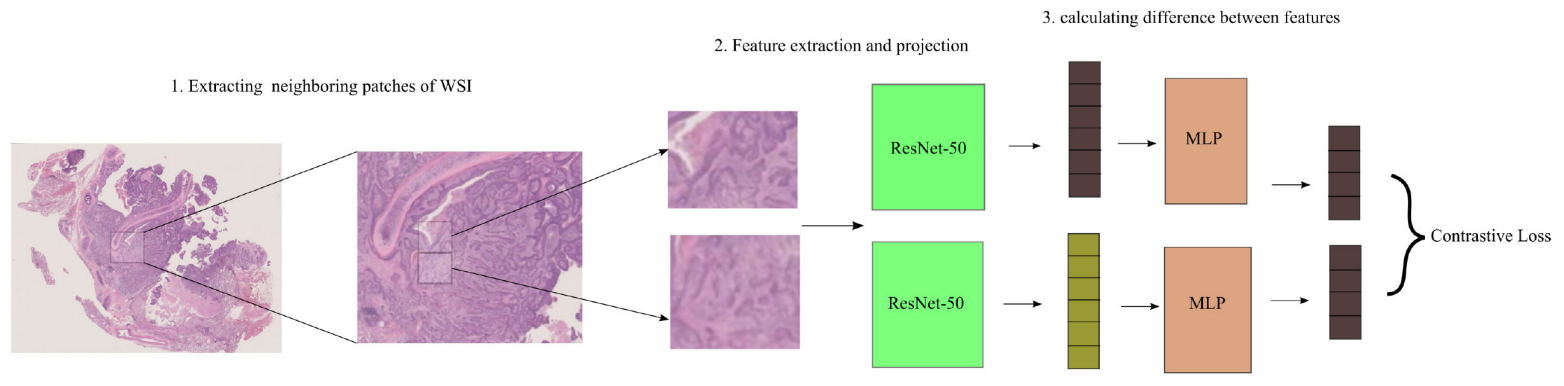
SimCLR training and inference. Two augmentations are done on a tile during training. Two augmentations of the same tile are supplied to a pre-trained ResNet-50 on ImageNet with an additional projection head. ResNet-50’s final convolutional block and projection head involves minimizing the contrastive across tiles. Features are retrieved from the refined ResNet-50 during inference. In the neighbor attention transformer module, patch distances are determined.

To estimate attention weights across instances that exhibit identical feature representations, we present the use of self-supervised contrastive learning. In this study, we focus on SimCLR (33), a widely recognized self-supervised learning system. In **Figure 2** SimCLR facilitates the acquisition of semantically meaningful feature representations by decreasing the dissimilarity between many augmented iterations of identical picture data.

After partitioning the segmented tissue region into tiles, we employ two distinctively enhanced variations of the identical tile as an input to an instance-level feature encoder denoted as *F*(*x*), which is built using a ResNet-50 architecture.

In the NATMIL framework, the last step involves the utilization of a projection head. This projection head is implemented as a multi-layer perceptron (MLP) containing two hidden layers. Its purpose is to transform the feature representations into a distinct space where a contrastive loss function is subsequently applied. During the training process, the feature representations zi and zj, which correspond to both viewpoints of the same tile that are differently augmented and correlated, are utilized in order to decrease adjusted temperature-scaled cross entropy as specified by Equation 1.


1 $$L\left(z_{t},z_{s}\right)=l\left(z_{t},q_{s}\right)+l\left(z_{s},q_{t}\right)$$

The function sim(.) represents cosine similarity, *τ* represents the variable temperature, and 1[k=i]∈0,1 is the value of a function that evaluates to 1 only if k = i.

$H=\left\{h_{1},\cdots ,h_{i},\cdots h_{N}\right\},h_{i}\in {\mathbb{R}}^{n\times d}$ of each WSI is generated using the ResNet-50 network as the base encoder, whereas n is the quantity of tiles and d is the embedding dimension.

### 3.2 Neighborhood Attention Transformer module

To encode the feature embeddings of the individual tiles, we utilize a transformer, *T*, layer to aggregate the feature embeddings $\;H=\left\{h_{1},\cdots ,h_{i},\cdots ,h_{N}\right\}$ , $h_{i}\in R^{n\times d}$ , where d is the embedding dimensions of a tile, *n* is the number of tiles inside a WSI, and *N* is the number of WSIs.

In this study, we propose the incorporation of a novel mechanism known as Neighborhood Attention (NA). We define attention weights for the *i*-th input with neighborhood size k , $A_{i}^{k}$ , in Equation 2 as the dot product of the *i*-th input’s query projection and its k nearest neighboring key projections. Given an input $X\in R^{n\times d}$ , which is a matrix whose rows are *d*-dimensional token vectors, and *X*’s linear projections, Q,K, and V , and relative positional biases  B(i,j) .


2 $$A_{i}^{k}=\left[\begin{array}{c} Q_{i}K_{\rho_{1}\left(i\right)}^{T}+B_{i,\rho_{1}\left(i\right)}\\ Q_{i}K_{\rho_{2}\left(i\right)}^{T}+B_{i,\rho_{2}\left(i\right)}\\ \cdot \\ \cdot \\ \cdot \\ Q_{i}K_{\rho_{k}\left(i\right)}^{T}+B_{i,\rho_{k}\left(i\right)} \end{array}\right]$$

Next, in Equation 3 we define $V_{i}^{k}$ , the adjacent values, as a matrix whose rows are the k nearest neighboring value projections of the *i*-th input:


3 $$V_{i}^{k}=\left[V_{\rho_{1}\left(i\right)}^{T},V_{\rho_{2}\left(i\right)}^{T},\cdots ,V_{\rho_{k}\left(i\right)}^{T}\right],$$

Next, we define attention for the *i*-th token with neighborhood size k as follows:


4 $$NA_{k}\left(i\right)=softmax\left(\frac{A_{i}^{k}}{\sqrt{d}}\right)V_{i}^{k}\;$$

with the scaling parameter denoted by $\sqrt{d}$ as shown in Equation 4. For each pixel in the feature map, this process is repeated.

**Figure 3:**
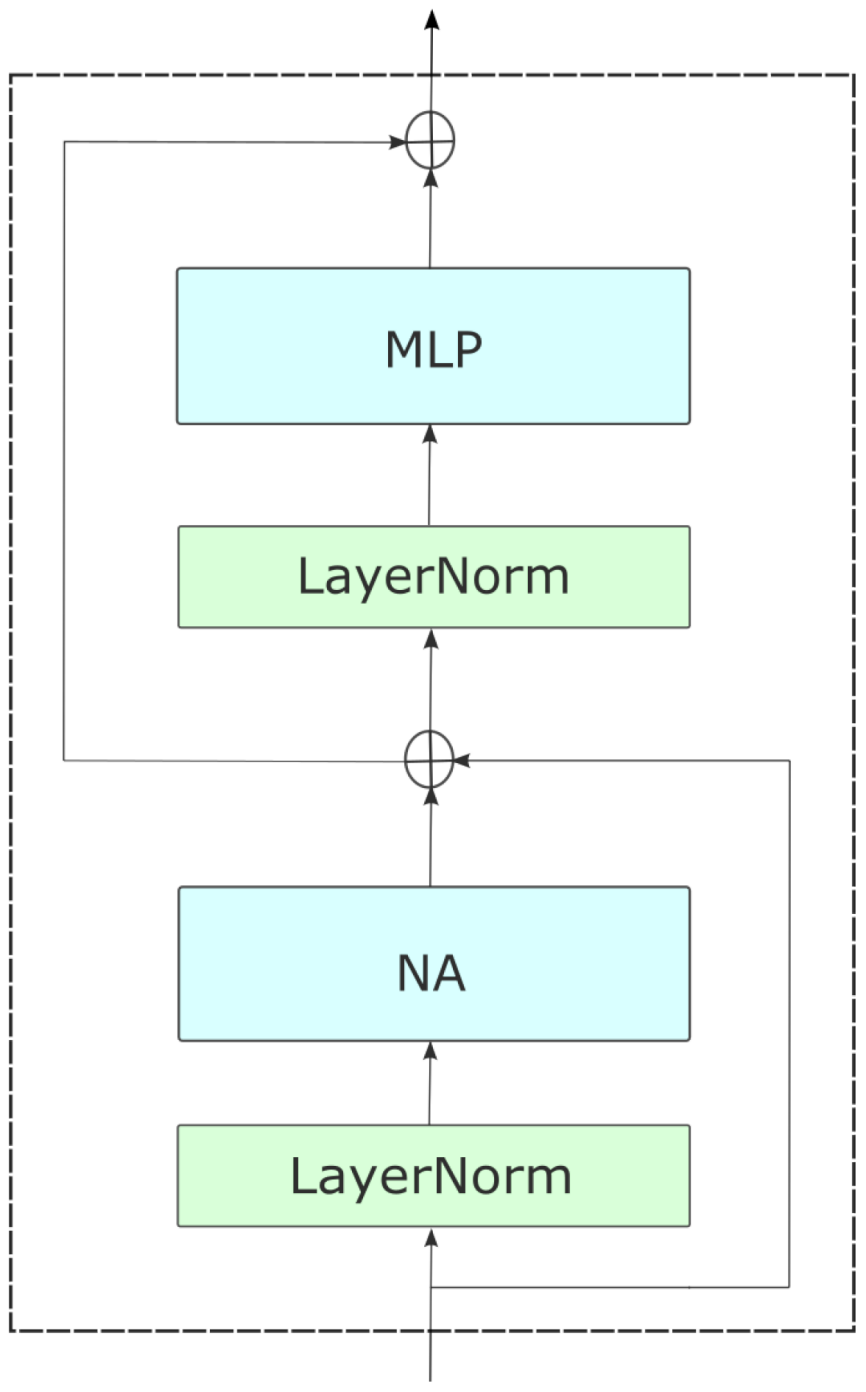
An overview of NAT, with its hierarchical design. The model begins with a convolutional downsampler and progresses through four successive stages containing numerous NAT Blocks, which are transformer-like encoder layers. The layers consist of a multi-headed neighborhood attention (NA), multi-layered perceptron (MLP), Layer Norm (LN) before each module, and skip connections. Between stages, feature maps are downsampled to half their spatial size and twice in depth.

With two consecutive 3 × 3 convolutions and 2 × 2 strides, NAT embeds inputs into a spatial size that is one-fourth that of the input as shown in **Figure 3**. This approach bears resemblance to employing a patch and embedding layer that consists of 4 × 4 patches. However, it diverges by employing overlapping convolutions instead of non-overlapping ones, thereby introducing valuable inductive biases. However, the utilization of overlapping convolutions would result in an escalation of expenses and an increase in the number of parameters due to the implementation of two convolutions. Nevertheless, we address this issue by reconfiguring the model, achieving an improved trade-off. With the exception of the last level, all four NAT levels are followed by a downsampler. Downsamplers double the number of channels while halving the spatial size. Instead of the 2 × 2 non-overlapping convolutions that Swin employs (patch merging), we employ 3 × 3 convolutions with 2 × 2 strides. As a result of the tokenizer’s fourfold downsampling, our model generates feature maps with sizes of $\frac{h}{4}\times \frac{w}{4},\frac{h}{8}\times \frac{w}{8},\frac{h}{16}\times \frac{w}{16},\frac{h}{16}\times \frac{w}{16}$ , The motivation for this shift stems from the success of previous CNN structures, which has since led to the development of various hierarchical attention-based approaches, like PVT (34), ViL (28), and Swin Transformer (29). Furthermore, Layer-Scale [29] is employed to provide stability in larger variations. **Figure 1** presents a visual representation of the entire network structure.

### 3.3 Feature aggregation

Aggregate WSI representation $g\in R^{1\times d}$ is adaptively calculated as a weighted average of individual value vectors, each weighted by Equation 5 its attention score in Equation 6.


5 $$g=\sum_{i=1}^{N}a_{i}\left(g_{i}+t_{i}\right)$$

such that


6 $$a_{i}=\frac{expw^{T}\left(tanh\left(Vt_{i}^{T}\right)⊙sigm\left(Ut_{i}^{T}\right)\right)}{\sum_{j=1}^{K}expw^{T}\left(tanh\left(Vt_{i}^{T}\right)⊙sigm\left(Ut_{i}^{T}\right)\right)}$$

The learnable parameters in this context are denoted as  U,V, and w . The symbol ⊙ represents element-wise multiplication. The function sigm() refers to the sigmoid non-linearity, whereas tanh() represents the hyperbolic tangent function.

At last, the classifier layer assigns each slide a score $W_{c}\in {ℛ}^{c\times d}$


7 $$y_{pred}=W_{c}g^{T}$$

where c is the total number of classes mentioned in Equation 7. Finally, a classification score is generated by using the representation learned from the well-attended patches to minimize a cross-entropy loss.

## 4 Experiments

### 4.1 Datasets

We conducted several tests using the Camelyon and TCGA-NSCLC datasets, both of which are widely utilized and publicly available. The Camelyon dataset stands out as a particularly significant open resource for studying breast cancer.

Among the largest public breast cancer datasets is Camelyon16 (35). It comprises a training set of 270 annotated biopsy slides and an official test set of 129 slides from Radboud University Medical Center and University Medical Center Utrecht in the Netherlands.

The TCGA-NSCLC dataset encompasses two distinct subtypes of non-small-cell lung cancer: lung squamous cell carcinoma (TGCA-LUSC) and lung adenocarcinoma (TCGA-LUAD). For LUAD, a total of 541 slides from 478 patients were obtained, while for LUSC, 512 slides from the same 478 cases were collected.

### 4.2 Baseline model

We evaluated the performance of our neighborhood pooling technique through a comparative analysis with classic pooling operators like Mean-pooling and Max-pooling, and various state-of-the-art Multiple Instance Learning (MIL) (36) methods. These methods include AB-MIL (37), CLAM-SB, CLAM-MB (15), MI Net, MIL-RNN (11), TransMIL (24), and DTFT-MIL (38).

The AB-MIL model incorporates attention mechanisms based on the specific attributes of each individual tile. In contrast, the CLAM-SB and CLAM-MB models also utilizeattention pooling operators similar to AB-MIL but are further enhanced by an auxiliary clustering layer. MI Net employs both max pooling and mean pooling techniques to generate the WSI-level embedding. On the other hand, the MIL-RNN model is an aggregation model that utilizes a recurrent neural network. TRANS-MIL utilizes a transformer-based aggregator, while DTFT-MIL employs the class activation map to calculate the positive probability of an instance within the AB-MIL framework.

### 4.3 Implementation

The tissue area was extracted from each slide using the publicly accessible WSI-preprocessing toolkit developed by (15). Subsequently, this region was divided into non-overlapping patches of size 256 × 256 at a magnification of ×20. It is important to note that variations in parameters during the feature extraction process may result in different training and test sets, potentially leading to varied model performance outcomes. Disseminating the extracted features allows other researchers to utilize the same dataset for training and evaluating their models, facilitating the comparison of different methodologies.

In our pipeline, the Neighborhood Attention Transformer component incorporated Swin’s (29) training configuration module, enabling the implementation of learning rate, iteration-wise cosine schedule, and other hyperparameters. The results are presented below.

## 5 Results

The outcomes of employing the NATMIL methodology for the classification of WSIs in the Camelyon16 and TCGA-NSCLC datasets are displayed in **Tables 1**, **2**. All tests in this study evaluate the performance using three metrics: the area under the receiver operating characteristic curve (AUC), the slide-level accuracy (ACC) with a threshold of 0.5, and the macro-averaged F1 score. These processes facilitated an acceptable evaluation across multiple techniques and datasets of varying sizes (39).

**Table 1:** Performance comparison of NATMIL against various baselines on the Camelyon16 datasets.

Method	ACC	F1	AUC
ABMIL-GATED	0.871 ± 0.025	0.842 ± 0.017	0.910 ± 0.027
MIL-RNN	0.872 ± 0.014	0.852 ± 0.016	0.921 ± 0.027
CLAM-SB	0.879 ± 0.023	0.862 ± 0.020	0.926 ± 0.021
CLAM-MB	0.882 ± 0.026	0.868 ± 0.031	0.927 ± 0.011
TRANSMIL	0.884 ± 0.013	0.869 ± 0.021	0.930 ± 0.013
DTFT-MIL	0.885 ± 0.013	0.871 ± 0.031	0.933 ± 0.021
**NATMIL**	0.896 ± 0.013	0.872 ± 0.015	0.940 ± 0.027

**Table 2:** Performance comparison of NATMIL against various baselines on the TCGA-NSCLC datasets.

Method	ACC	F1	AUC
ABMIL-GATED	0.859 ± 0.013	0.852 ± 0.017	0.880 ± 0.057
MIL-RNN	0.864 ± 0.023	0.862 ± 0.031	0.890 ± 0.038
CLAM-SB	0.839 ± 0.011	0.862 ± 0.023	0.897 ± 0.026
CLAM-MB	0.847 ± 0.009	0.866 ± 0.061	0.9320 ± 0.027
TRANSMIL	0.865 ± 0.020	0.872 ± 0.061	0.940 ± 0.027
DTFT-MIL	0.879 ± 0.022	0.862 ± 0.054	0.920 ± 0.027
**NATMIL**	0.881 ± 0.0303	0.882 ± 0.017	0.940 ± 0.027

**Figure 4:**
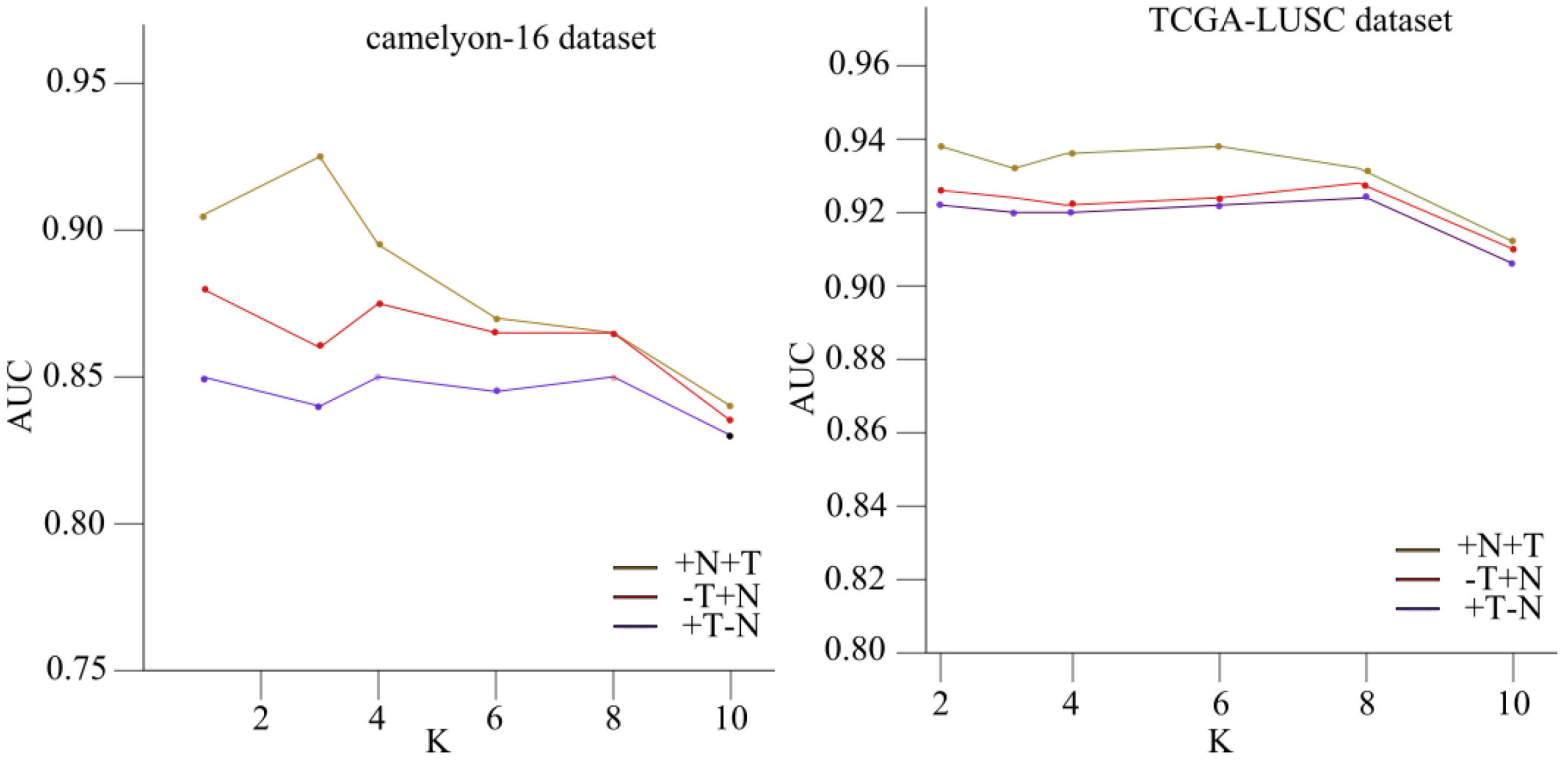
The link between the hyperparameter “k” and the corresponding area under the receiver operating characteristic curve (AUC) values for the Camelyon16 and TCGA-NSCLC histopathology datasets.

The results presented in the tables are further elucidated in **Figure 4**, which illustrates the relationship between the hyperparameter “ k ” and the corresponding area under the receiver operating characteristic curve (AUC) values for the Camelyon16 and TCGA-NSCLC histopathology datasets.

The figure demonstrates the impact of varying the neighborhood size “ k ” on the performance of the NATMIL model. For lower values of “ k ” (i.e., k∈2,3,4 ), the model exhibits similar behavior across both datasets, performing consistently well under identical experimental conditions. This consistency is expected, as nearby tiles convey significant information regarding the risk of a tile being malignant. However, as the value of “k” increases, there is a progressive decline in the model’s performance, except for a notable improvement when “ k ” equals 8.

This observed phenomenon can be attributed to recurring patterns within tumors, occurring at intervals of approximately eight tiles. Thus, the significance of employing models capable of capturing both local adjacent information and overall trends in the biopsy is underscored. It is also noteworthy that selecting either “ k=4 ” or “ k=8 ” consistently yields satisfactory outcomes due to the spatial configuration of tiles and their neighboring elements, reminiscent of a grid-like topology.

NATMIL surpasses all previous MIL models in terms of accuracy and AUC on the Camelyon16 cancer dataset. Notably, within the Camelyon16 dataset, tumor cells might constitute a mere 5% of the WSI. The occurrence of tumor cells in tissue samples is frequently observed at a low frequency, especially in metastatic locations, where tumor cells are distributed among extensive areas of normal cells (40). Therefore, the NATMIL model, which utilizes a local neighborhood analysis to readjust attention coefficients, demonstrated superior efficacy in detecting medically significant, sparsely distributed malignant spots compared to alternative models. The performance of NATMIL on the Camelyon16 dataset exhibited substantial superiority over the other baselines. The NATMIL model demonstrates a statistically significant improvement of at least 1.5% in terms of AUC compared to other currently available models.

We present the experimental results of the proposed methods on CAMELYON-16 and TCGA lung cancer dataset in comparison to the following baselines methods: i) classic AB-MIL; ii) RNN-based RNN-MIL; iii) attention-based CLAM-SB, CLAM-MB; and iv) transformer-based MIL, Trans-MIL.

For CAMELYON-16, most slides contain only small portions of tumor over the whole tissue region. The proposed NATMIL methods with different features have outperformed other existing MIL methods except Trans-MIL, which used a transformer-based aggregator, while Trans-MIL is significantly larger in model size and computational complexity. The NATMIL achieves significant performance at AUC of 0.7% better than DTFT-MIL, as the model used different feature distillations.

For TCGA lung cancer, the proposed methods also achieve leading performances, with NATMIL obtaining the best AUC value of 94.2%. Due to the significantly larger tumor regions in positive slides, even RNN and DTFT-based MIL methods perform well on the TCGA lung cancer dataset resulting in less obvious superiority of the proposed methods over other existing methods. In comparison, for the much more challenging dataset CAMELYON-16, the proposed method present robustness to the situation of small portions of tumor regions in positive slides.

In the TCGA-NSCLC dataset, it was observed that NATMIL had superior performance compared to the other baselines that were taken into consideration. The max-pooling approach, which employs the max operator as an aggregation function, demonstrated superior performance compared to other methods. The remarkable efficacy of max pooling on this dataset can be attributed to the observation that tumor cells constitute approximately 80% of the WSI in the TCGA-NSCLC dataset. The probability of accurately labeling distinct malignant cells is significantly elevated.

### 5.1 Ablation study

Our ablation investigation examined the efficacy of the Neighborhood Attention (NA) design block and the surrounding attention module. We tested how changing the neighborhood size *k* affected the efficiency of our NATMIL model. As shown in **Figure 4**, we observed that for low values of *k* (i.e., *k* ∈ 2,3,4), the model behaved similarly after being trained under identical experimental conditions. This consistency makes sense, given that nearby tiles convey the most significant information regarding the risk of a tile being malignant. The desirability of robustness in the selection of *k* stems from the time-consuming nature of hyperparameter adjustment. However, as the value of *k* increased, there was a progressive decline in the model’s performance, except for a notable improvement when *k* equaled 8.

The observed phenomenon can be attributed to the emergence of recurring patterns within tumors, occurring at intervals of approximately eight tiles. This underscores the significance of employing models capable of capturing both local adjacent information and overall trends in the biopsy. It was also noted that the selection of either *k* = 4 or *k* = 8 consistently yielded appropriate outcomes due to the spatial configuration of tiles and their neighboring elements, which exhibit characteristics reminiscent of a grid-like topology.

We examined the impact of our NAT design, which includes convolutional downsampling and a deeper-thinner architecture. To evaluate its effectiveness, we conducted an ablation study comparing models utilizing self-attention and shifted window self-attention. The model was gradually transformed into NAT, and the outcomes are displayed in **Table 3**. The initial step

**Table 3:** Accuracy performance of different attention and convolutions on the TCGA-NSCLC datasets.

Attention	Downsampler	#of layers	#of heads	#MLP Ratio	AUC
Self-Attn	Patch	2, 4, 6, 2	3	4	0.9061
Window self-Attn	Conv	2, 4, 6, 2	3	4	0.9131
Neighbor Attn	Conv	3, 4, 18, 5	2	3	0.9210
Convolution	Conv	3, 4, 18, 5	2	3	0.9127

involved substituting the patched embedding and patched merge techniques with the overlapping convolution design employed in the Neighborhood Attention Transformer (NAT) model. This led to an increase in accuracy of approximately 0.5%. Upon implementing the second phase of reducing the model size and computational load by increasing its depth and reducing its width, an approximate improvement in accuracy of 0.9% compared to the initial step was observed. As a result, a minor decrease in accuracy was observed. Nevertheless, by substituting Window-Shifted Attention and Self-Window-Shifted Attention with our Neighborhood Attention, a notable enhancement of 0.5% in accuracy was observed.

Additionally, we conducted a kernel size investigation as shown in **Table 4**. The experiment involved varying kernel sizes from 3×3 to 9×9 in order to examine the impact on the performance of our model.

**Table 4:** Performance comparison of NATMIL with different kernel size on TCGA-LUSC datasets.

Kernel size	ACC	AUC
3×3	0.8900 ± 0.0137	0.9260 ± 0.3206
5×5	0.8810 ± 0.9938	0.9263 ± 0.2637
7×7	0.8920 ± 0.0545	0.9304 ± 0.5445
9×9	0.8980 ± 0.0131	0.9401 ± 0.1238

## 6 Conclusion

In this paper, we present the first effective and scalable sliding window attention technique for vision, called Neighborhood Attention. The first aggregation method employs the independence assumption to provide an attention score for each tile in the picture, whereas the second uses vision transformers to produce an attention score that accounts for the correlation between tiles.

To re-adjust the estimated attention ratings based on the similarities they share, we have introduced NATMIL, a unique MIL vision transformer-based method that considers the interdependence of nearby tiles in a histopathological image. By leveraging the pathologists’ existing slide-level labeling, NATMIL improves performance, reduces their burden, and makes more data available.

## Data Availability

The datasets presented in this study can be found in online repositories. The names of the repository/repositories and accession number(s) can be found below: https://portal.gdc.cancer.gov/.
